# TRPV4 related skeletal dysplasias: a phenotypic spectrum highlighted byclinical, radiographic, and molecular studies in 21 new families

**DOI:** 10.1186/1750-1172-6-37

**Published:** 2011-06-09

**Authors:** Elena Andreucci, Salim Aftimos, Melanie Alcausin, Eric Haan, Warwick Hunter, Peter Kannu, Bronwyn Kerr, George McGillivray, RJ McKinlay Gardner, Maria G Patricelli, David Sillence, Elizabeth Thompson, Margaret Zacharin, Andreas Zankl, Shireen R Lamandé, Ravi Savarirayan

**Affiliations:** 1 Genetic Health Services Victoria and Murdoch Childrens Research Institute, Parkville, Victoria, Australia; 2 Department of Clinical Pathophysiology, University of Florence and Meyer Children's Hospital Genetics Unit, Florence, Italy; 3 Northern Regional Genetic Services, Auckland Hospital, Auckland, New Zealand; 4 Department of Genetic Medicine, University of Sydney, New South Wales, Australia; 5 Paediatrics, Women's and Children's Hospital, Adelaide, Australia; South Australian Clinical Genetics Services, SA Pathology (at Women's and Children's Hospital), Adelaide, Australia; 6 Paediatrician Midcentral District Heath Board, Palmerston North, New Zealand; 7 Genetics and Metabolic Medicine, Hospital for Sick Children, Toronto, Canada; 8 Genetic Medicine, St Mary's Hospital, Manchester, UK; 9 Biologia molecolare clinica e citogenetica, Diagnostica e Ricerca, San Raffaele SPA, Milan, Italy; 10 South Australian Clinical Genetics Service, South Australian Pathology, Women's & Children's Hospital, North Adelaide, South Australia, Australia; 11 Department of Endocrinology and Diabetes, Royal Children's Hospital, Parkville, Victoria, Australia; 12 Genetic Health Queensland, Royal Brisbane and Women's Hospital, Brisbane, Australia; 13 UQ Centre for Clinical Research, The University of Queensland, Brisbane, Australia; 14 Murdoch Childrens Research Institute, Royal Children's Hospital, Parkville, Victoria, Australia

**Keywords:** TRPV4, Metatropic Dysplasia (MD), Autosomal Dominant Brachyolmia (ADBO), Spondilometaphyseal Dysplasia Kozlowski Type (SMDK)

## Abstract

**Background:**

The *TRPV4 *gene encodes a calcium-permeable ion-channel that is widely expressed, responds to many different stimuli and participates in an extraordinarily wide range of physiologic processes. Autosomal dominant brachyolmia, spondylometaphyseal dysplasia Kozlowski type (SMDK) and metatropic dysplasia (MD) are currently considered three distinct skeletal dysplasias with some shared clinical features, including short stature, platyspondyly, and progressive scoliosis. Recently, *TRPV4 *mutations have been found in patients diagnosed with these skeletal phenotypes.

**Methods and Results:**

We critically analysed the clinical and radiographic data on 26 subjects from 21 families, all of whom had a clinical diagnosis of one of the conditions described above: 15 with MD; 9 with SMDK; and 2 with brachyolmia. We sequenced *TRPV4 *and identified 9 different mutations in 22 patients, 4 previously described, and 5 novel. There were 4 mutation-negative cases: one with MD and one with SMDK, both displaying atypical clinical and radiographic features for these diagnoses; and two with brachyolmia, who had isolated spine changes and no metaphyseal involvement.

**Conclusions:**

Our data suggest the *TRPV4 *skeletal dysplasias represent a continuum of severity with areas of phenotypic overlap, even within the same family. We propose that AD brachyolmia lies at the mildest end of this spectrum and, since all cases described with this diagnosis and *TRPV4 *mutations display metaphyseal changes, we suggest that it is not a distinct entity but represents the mildest phenotypic expression of SMDK.

## Background

Metatropic Dysplasia (MD, OMIM 156530) is a severe bone dysplasia, observable in the perinatal period, first described by Maroteaux et al in 1966. Derived from ancient Greek μετα meaning "with" and τροπη meaning "change"; the name refers to the main clinical feature of the condition, which changes from the newborn period, with long trunk and short limbs, to childhood, when, due to progressive spinal changes, it becomes a short trunk dwarfism. The main abnormalities occur in the vertebral bodies and the metaphyseal portions of the long bones, and the clinical and radiological diagnostic features are well described in the literature [1]. Some authors have suggested genetic variability in MD, with an autosomal dominant type and a more severe (lethal) autosomal recessive type,[2] but Camacho et al (2010) demonstrated *TRPV4 *mutations in both lethal and milder types of MD [3].

Spondylometaphyseal Dysplasia Kozlowski Type (SMDK, OMIM 1842522) was first described by Kozlowski, Maroteaux and Spranger in 1967 [4] and it is the best-characterized SMD. It is an autosomal dominant (AD) condition, with postnatal short stature, and progressive spine deformities. The typical radiographic features are platyspondyly and medially placed vertebral pedicles in the spine; metaphyseal irregularities, especially in the proximal femur; abnormal acetabula and delayed carpal bone age [5]. In 2009 Krakow et al demonstrated that SMDK, like MD, is caused by mutations in the gene encoding TRPV4 [6].

Brachyolmia was a term coined in 1969 by Maroteaux for a group of bone dysplasias which displayed short trunk short stature, with radiological involvement of the spine and no significant abnormalities of the long bones [7]. Twenty years later this group was classified into 4 different types: Hobaek, Toledo and Maroteaux, with autosomal recessive inheritance, and an autosomal dominant type of brachyolmia (OMIM 113500). The Toledo type is distinguished by corneal opacities, but it is otherwise identical to the Hobaek type; Maroteaux type was subsequently shown to also have epiphyseal involvement and it is classified in a different group in the nosology of skeletal disorders published in 2006 [8]. AD brachyolmia always showed slightly different features from the recessive types, with a much more severe involvement of the spine, progressive scoliosis and abnormalities in the cervical vertebrae too [7]. In 2008 Rock et al demonstrated that AD brachyolmia is caused by gain of function mutations in the *TRPV4 *gene [9].

TRPV4 is a cation channel, non-selectively permeable to calcium, encoded by a gene on chromosome 12. It is widely expressed and involved in many different physiological processes through responses to several different stimuli [10]. The full understanding of all the functions of this complicated protein is still elusive, but new data constantly emerge, explaining some of the features of this channel. The fact that mutations in the *TRPV4 *gene cause bone dysplasias is consistent with the channel being expressed in osteoblasts and ostoclasts [11] and having an important role in cartilage, since it is expressed in the early stages of chondrocyte differentiation [12] and modulates *SOX9 *expression in cell culture [13].

More than 30 different *TRPV4 *mutations underlying these three skeletal dysplasias have been reported in the literature to date, mutational hotspots have been identified, and some tentative genotype-phenotype correlations made. We confirm some of the published data and report 5 new mutations, including the first duplication/insertion. Our data also call into question the genotype-phenotype correlations previously reported.

## Materials and methods

The study was approved by the Royal Children's Hospital (Melbourne, Australia) human ethics research committee (project reference 20045). All patients gave their informed consent for the study.

**Figure 1:**
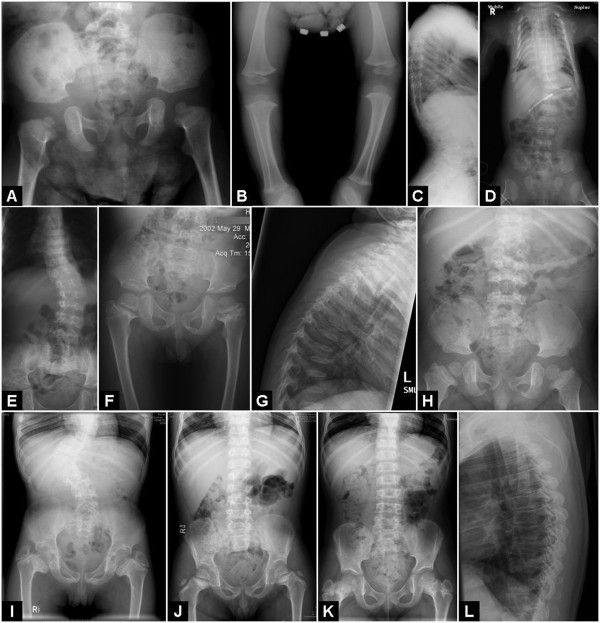
**X-rays of some of the cases described**. A, B, C, pelvis, legs and lateral spine of case 5 (MD-SMDK bridging phenotype); D, case 8 (MD); E, F, AP spine and pelvis of case 16 (SMDK); G, H, lateral spine and pelvis of patient 18 (SMDK); I, AP spine and pelvis of case 19 (SMDK); J, AP spine and pelvis of case 20 (SMDK); K, AP spine and pelvis of case 21 (SMDK); L, lateral spine and pelvis of case 26, who had some minor spinal changes and no metaphyseal involvement.

Clinical assessment of the patients was done by participating clinicians; the radiological features were reviewed by bone dysplasia experts (RS, DS). The key diagnostic criteria for MD were severe platyspondyly, progressive spinal deformity, halberd-shaped pelvis and dumbbell-shaped diaphyses. SMDK was characterized by platysopndyly, overfaced pedicles, flared metaphyses of the long bones and flat acetabula. AD brachyolmia was considered, based on the literature [8], as a condition affecting only the spine, with platyspondyly and vertebral abnormalities, but no metaphyseal changes (Figure 1).

DNA was extracted from whole blood samples with standard methods, using a PureLink™ Genomic DNA Mini Kit (Invitrogen). The sixteen exons and flanking intronic sequences of *TRPV4 *(NCBI Reference Sequence NM_021625.4) were PCR amplified from 25 ng of DNA using previously described primer sequences and conditions (9). PCR products were then treated with ExoSAP-IT^® ^(Affymetrix^®^) and sequenced with a BigDye Terminator v3.1 Cycle Sequencing Kit (Applied Biosystems) using the PCR primers. The products were separated on a 3130 Genetic Analyzer (Applied Biosystems). Each mutation was confirmed on a second PCR product, and the parents were tested where possible. If the parents were not available a panel of 90 control DNAs obtained with approval from the Australian Red Cross Blood Service Ethics Committee was tested.

For the novel missense variants we also checked that the involved amino acids were conserved among diverse species on PANTHER (http://www.pantherdb.org) and performed in silico analysis with SIFT (http://sift.jcvi.org) and POLYPHEN (http://genetics.bwh.harvard.edu/pph).

## Results

We analysed DNA samples from 26 subjects: 15 had a diagnosis of MD; 9 had SMKD; and 2 had brachyolmia. We found mutations in 22 patients from 17 families: among our patients there are familial cases of both MD (patients 2 and 3 and patients 5, 6, and 7) and SMDK (patients 19, 20, and 21). We found 9 mutations: 4 had been previously described, while 5 were novel (Table 1). All the mutations, other than c.1566_68dup (p.L523dup), were missense mutations. The amino acids involved in new mutations were conserved among species on PANTHER (Additional file 1). SIFT and POLYPHEN both predicted Y591C, R594H, R594S, P799L, P799S were deleterious but gave discordant results for K407E and F617L. The mutation Q239H was not considered deleterious by either SIFT or POLYPHEN, but we evaluated the fact that unaffected parents did not carry the same mutation as more significant: more functional studies are necessary to confirm the pathogenic role of this variant.

**Table 1:** Patients included in the study, clinical diagnosis and mutations

*Patient*	*Family*	*Diagnosis*	*Mutation*	*Exon*
1	1	MD	c.1219A>G; p.K407E*	7
2	2	MD	c.2396C>T; p.P799L	15
3	2	MD	c.2396C>T; p.P799L	15
4	3	MD	c.2396C>T; p.P799L	15
5	4	MD/SMDK	c.1781G>A; p.R594H	11
6	4	MD/SMDK	c.1781G>A; p.R594H	11
7	4	MD/SMDK	c.1781G>A; p.R594H	11
8	5	MD	c.2395C>T; p.P799S	15
9	6	MD	c.1780C>A; p.R594S*	11
10	7	MD	c.1781G>A; p.R594H	11
11	8	MD	c.1781G>A; p.R594H	11
12	9	MD	c.2396C>T; p.P799L	15
13	10	MD	c.2396C>T; p.P799L	15
14	11	MD	c.717G>C; p.Q239H*	5
15	12	SMDK	c.1781G>A; p.R594H	11
16	13	SMDK	c.1781G>A; p.R594H	11
17	14	SMDK	c.1566_68dup; p.L523dup*	9
18	15	SMDK	c.1851C>A; p.F617L	12
19	16	Brachyolmia	c.1772A>G; p.Y591C*	11
20	16	SMDK	c.1772A>G; p.Y591C*	11
21	16	SMDK	c.1772A>G; p.Y591C*	11
22	17	SMDK	c.1781G>A; p.R594H	11
23	18	MD	negative	-
24	19	SMDK	negative	-
25	20	Brachyolmia	negative	-
26	21	Brachyolmia	negative	-

* indicates novel mutations not previously described in the literature

Clinical features of cases not previously described in the literature are described in Table 2.

**Table 2:** Clinical and radiographic features of all patients, a part from the ones already described in the literature (1)

PATIENT		1	5	6	7	8	12	13	15	16	17	18	19	20	21	22	23	24	25	26
**DIAGNOSIS**		MD	MD	MD	MD	MD	MD	MD	SMDK	SMDK	SMDK	SMDK	SMDK	SMDK	SMDK	SMDK	MD	SMDK	ADB	ADB
**STATURE**	SHORT	+	+	+	+	-	+	+	+	+	-	+	+	-	-	+	+	+	+/-	+
	SHORT TRUNK	+	+	+	+	-	+	+	+	+	+/-	+	+	+/-	+/-	+	N/A	-	+	+/-
**SPINE**	(KYPHO)SCOLIOSIS	+	+	+	+	+	+	+	+	+	+/-	-	+	+/-	-	+	+	+/-	-	-
	PLATYSPONDYLY	+	+	+	+	-	+	+	+	+	+	+	+	+	+	+	N/A	-	+	+/-
	OVERFACED PEDICLES	-	+/-	+/-	+/-	-	-	-	+	+	+	+	+	+	+	+	N/A	-	-	-
**PELVIS**	HALBERD SHAPED	+	-	-	-	+	+	+	-	-	-	-	-	-	-	-	N/A	-	-	-
**METAPHYSEAL** **INVOLVMENT**	SHORT FEMORAL NECKS	+	+	+	+	+	+	+	+	+	+	+	+	+	+	+	N/A	+	-	-
	FLARED METAPHYSES	+	-	+/-	+/-	++	+	+	-	-	+/-	-	-	-	-	-	N/A	-	-	-
**RESPIRATORY** **PROBLEMS**		+	-	-	-	++	+	+	-	-	-	-	-	-	-	-	+/-	-	-	-

MD = Metatropic Dysplasia; SMDK = Spondylometaphyseal Dysplasia Kozlowski type; ADB = Autosomal Dominant Brachyolmia; the feature is considered as present and severe (++); present (+); mildly present (+/-); absent (-); data not available (N/A)

Among the patients with MD, all but one (case 23) had a clear diagnosis of MD and seven of these cases had been already described in the literature (cases 2,3,4,9,10,11,14) [1]. There were 2 cases (patients 8 and 14) with a very severe/lethal phenotype.

Patient 8 was born at term with normal birth parameters, but immediately hospitalized because of skeletal malformations and respiratory problems. She had very severe cervical instability and died at 4 1/2 months of age after a possible episode of medullary compression/respiratory decompensation. The mutation we found in her (P799S) has already been described as a typical MD mutation and the codon is a mutational hotspot [14].

In patient 14, already described in the literature, a diagnosis of probable lethal bone dysplasia was made *in utero*, and the pregnancy was interrupted at 23 weeks of gestation: the post mortem examination led to the diagnosis of MD [1,15]. The *TRPV4 *mutation (Q239H) this patient had is novel and neither of the parents showed the same change. The analysis on PANTHER showed that the involved amino acid is conserved.

Amino acid P799 was mutated in 5 families (cases 2 and 3 are father and daughter) and all these patients had a typical MD phenotype. Also cases 9, 10 and 11 had a typical MD phenotype [1] and they all had changes to amino acid R594. We found the recurrent mutation R594H, commonly described in SMDK patients,[6,14] but also a new change, c.1780C>A (p.R594S) in subject 9. He has a very severe spinal phenotype for which he underwent several surgeries and which is the cause, in adulthood, of restrictive lung disease [1].

Cases 5, 6 and 7 are from the same family (mother and two sons): initially a diagnosis of MD was made, but after further reviewing the x-rays, we diagnosed a bridging phenotype between MD and SMDK given that some radiographic features in one sibling were more consistent with MD, whereas the other sibling had radiology most consistent with SMDK. This illustrates the degree of intrafamilial variability within this spectrum of disorders.

Case 1 was born at term with normal birth parameters after an uneventful pregnancy. In the first months of age she was repeatedly admitted to hospital because of recurrent respiratory infections: at 3 1/2 months the diagnosis of recurrent aspirations, in-coordinate swallowing, narrowing of the distal third of the trachea and tracheobronchomalacia was made; on this occasion an incidental finding of spinal abnormalities led to a skeletal survey which in turn enabled the diagnosis of MD. The mutation she carried (K407E) was novel. The mother did not show the same variant and the father's sample was not available; however the mutation was not found in 90 control individuals (180 chromosomes). PANTHER shows it is a conserved amino acid and SIFT considers the change "not tolerated". These findings led us to consider this a pathogenic variant.

Patients 15 and 16 had a diagnosis of typical SMDK, so the finding of the R594H mutation did not surprise us, since it had already been described as a recurrent mutation in SMDK [6].

In case 17 a diagnosis of SMDK was made when she was 2 years old: on x-rays she had platysponyly, metaphyseal flaring of the long bones, short femoral necks and a small sacrosciatic notch. The molecular analysis showed the first described insertion/duplication in the *TRPV4 *gene: c.1566_68dup (p.L523dup). The parents were analysed and the variant is *de novo*.

Patient 18 was born at term to non-consanguineous healthy parents and had normal birth parameters. His perinatal history was reported to be unremarkable and his motor development was normal (walking at 12 months of age). When he was 18 months old his mother started noticing some bowing of the lower limbs: at 2 years a skeletal survey was done, the x-rays showed spinal changes and metaphyseal involvement and a diagnosis of SMDK was made. The mutation he carries (c.1851C>A; p.F617L) has been previously described in a patient with mild MD [3].

Patient 19 was born after an uneventful pregnancy; his mother, maternal uncle, grandfather and greatgrandmother are all affected by short stature with spinal changes. The x-rays in different family members showed severe platyspondyly, overfaced pedicles in some family members and some also showed short femoral necks and coxa vara. Patient 19 was evaluated at 9 years of age: he then had a very severe kyphoscoliosis and a diagnosis of AD Brachyolmia was made, since there were no significant changes in the metaphyses. Four years later his sister (case 20) and half brother (case 21) were referred to the same bone dysplasia expert, who did not know about the kinship at the time he saw the x-rays, and made a diagnosis of SMDK. In this family the mutation c.1772A>G (p.Y591C) was found.

Four of the patients included in this study did not have *TRPV4 *mutations. Patients 23 and 24 did not have typical features of MD and SMDK respectively. Case 23 had some features of MD, like short stature, early scoliosis and a caudal appendage; but also had learning disabilities, recurrent knee dislocations, very hyperextensible joints and some dysmorphic features, and likely had a separate (undiagnosed) syndrome. Case 24 showed short femoral necks and scoliosis as a child, but also developed joint laxity, dysmorphic features and a short limb short stature instead of a short trunk short stature. Although considered atypical for MD, it is possible that there could be genetic (locus) heterogeneity in this condition with another (possibly pathway linked) gene responsible. Cases 25 and 26, diagnosed with brachyolmia, had only vertebral changes with absolutely no metaphyseal involvement. (Table 2)

## Discussion/Conclusions

We describe the clinical and molecular features of 26 subjects with *TRPV4*-related bone dysplasias (MD, SMDK, and brachyolmia). These data highlight that these chondrodysplasias constitute an allelic "family" in which there is a spectrum of overlapping severity ranging from lethal MD to mild AD brachyolmia, which we contend is the mildest phenotypic expression of SMDK, as all cases have radiographic evidence of metaphyseal involvement.

We found 9 mutations in 22 patients: 4 are previously described mutations, 5 are novel.

This study has also identified the first *TRPV4 *insertion: a de novo c.1566_68dup (p.L523dup) found in patient 17, who has a typical SMDK phenotype. The CCT insertion after the second base of codon 523 maintains the leucine at codon 523 and inserts an additional leucine codon (CCG) at this position. The sequence thereafter remains in-frame.

Two MD patients had a very severe/lethal phenotype: patient 8, with mutation P799S and patient 14, with mutation Q239H. Some authors [2] proposed different inheritance modes for MD, with a lethal, possibly recessive, type of MD, and a dominant "classic" MD. Others, however,[3] have shown *TRPV4 *heterozygous mutations in very severe/lethal MD.

Our data confirm dominant mutations underlie very severe/lethal MD. This information is crucial for genetic counselling and recurrence risk assessment as this disorder was previously considered a recessive trait with 25% recurrence risk.

The family with the Y591C mutation (pts 19,20,21) was particularly illustrative in the fact that the same bone dysplasia expert made different diagnoses in half brothers (AD brachyolmia/SMDK respectively), not knowing they came from the same family. These cases, and the fact that neither of the patients (24 and 25) with purely isolated spinal changes showed *TRPV4 *mutations, prompted us to critically review the AD brachyolmia phenotype. The first cases of brachyolmia with *TRPV4 *mutations reported in the literature showed, in association with the typical vertebral abnormalities, mild metaphyseal changes in the femoral heads and delayed bone age [9]. Dai et al. (2010) wrote about "two SMDK patients who did not show overt metaphyseal changes and were considered to be of intermediate severity between SMDK and Brachyolmia" [14]. Kozlowski questioned the existence of brachyolmia as a separate entity, saying that all patients with brachyolmia had at least some mild metaphyseal changes, with shortening of femoral necks representing the minimal diagnostic changes, and for that reason had to be considered as spondylometaphyseal dysplasia patients [16]. Given these observations and the data from our study, we propose that "true" brachyolmia, i.e. with changes limited only to the spine, is the recessive Hobaek type, while "autosomal dominant brachyolmia" should be considered as the mildest end of the spectrum of SMDK. SMDK cases with very mild metaphyseal changes have been described [17] as have metaphyseal irregularities (especially in the femurs) in AD brachyolmia [18].

As the molecular data on *TRPV4 *mutations are becoming more available, it is feasible to try to find valid genotype-phenotype correlations as it is increasingly evident that these *TRPV4 *skeletal dysplasias, have considerable phenotypic overlap with blurred boundaries.

Our data confirm that P799 and R594 are the most commonly mutated codons in the TRPV4 skeletal dysplasias; however, as others have reported [14], it is not always easy to make a definite association between a specific codon and a specific phenotype. Codon 799 was mutated in 6 subjects: this was already considered a mutational hotspot, and different amino acid changes had been described. All our patients with a mutation in codon 799 had MD, consistent with the reported experience [3,14]. Nine subjects had a mutation at codon 594: R594H is a recurrent mutation and the finding of a different amino acid change (R594S) confirms this codon as another mutational hotspot. In contrast to the published data, we found this mutation in MD subjects as well as in SMDK. We therefore suggest that the recurrent mutation R594H should be considered when an apparent bridging phenotype between SMDK and MD is observed.

For some of the mutations there seems to be a good genotype-phenotype correlation, based on the cases described in the literature, like P799L or F617L, which are associated respectively with classical MD and "mild" MD/"severe" SMDK. Our data show, however, that this is not true for all the mutations described and it is not even possible, at present, to find a true association between a specific protein domain and bone dysplasia disease severity. Mutations are spread throughout the protein and mutations causing MD, for example, are found in the ankyrin repeat domain as well as in the C-terminal domain.

Some of the mutations described in the literature have been tested functionally and found to be gain of function mutations. We know TRPV4 is a very complex protein and many authors have demonstrated with *in vitro *experiments that different changes at the same codon can affect the channel response to agonists differently [19]. In the last year the picture became even more complex when mutations in the TRPV4 protein were shown to cause not only the above-mentioned bone dysplasias but also neurologic conditions such as congenital distal Spinal Muscular Atrophy (SMA), scapuloperoneal SMA and hereditary motor and sensory neuropathy 2C (also known as Charcot-Marie-Tooth type 2C) [20-22]. Recently, other bone dysplasias have been associated with *TRPV4 *mutations too, like spondylo-epiphyseal dysplasia, Maroteaux type (pseudo-Morquio syndrome type 2) and parastremmatic dysplasia [23].

For a full understanding of the pathogenic mechanisms that lead from mutations in this complex gene to such a variety of different conditions, more functional data on the mutant channels is required as well as more information about how interactions between the TRPV4 channel and other proteins are disrupted in these conditions and how downstream physiological processes are perturbed.

**Figure 2:**
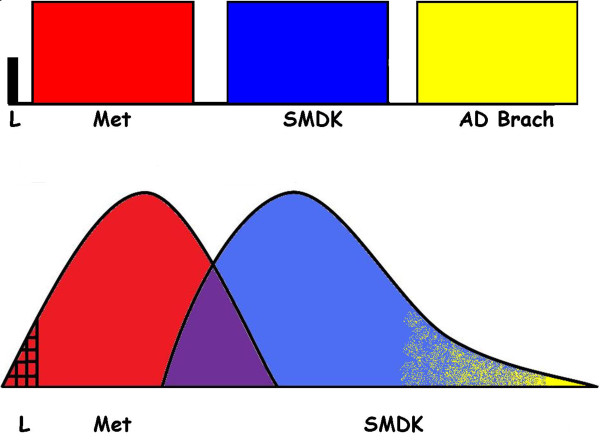
**Different perspectives in classification of the TRPV4 bone dysplasias**. On the top the classification into separate conditions; on the bottom the idea that lethal MD is the severe end of the MD spectrum; there is a region where the clinical features of MD and SMDK overlap; and AD brachyolmia is the mild end of the SMDK spectrum.

In conclusion, the data from this study strongly suggest that: a) the condition previously considered autosomal recessive, lethal MD represents the severe end of the MD phenotypic spectrum, as demonstrated by our findings and by previous data from the literature [3]; b) MD and SMDK, should be considered part of a *TRPV4 *bone dysplasia spectrum with significant overlapping clinical and radiographic features, even within families; c) AD brachyolmia is not a separate phenotypic entity and should be considered as the mildest expression of the SMDK spectrum, while AR brachyolmia has different radiographic features and, probably, a different genetic cause (Figure 2).

## Competing interests

The authors declare that they have no competing interests.

## Authors' contributions

EA planned and co-ordinated the study, wrote the manuscript, saw some of the patients and revised all the clinical data, carried out the molecular genetic studies. SA, MA, EH, WH, PK, BK, GMG, RJMKG, MGP, DS, ET, MZ, AZ gave important contribution to the clinical work, seeing patients, making diagnoses and collecting clinical data. SL planned and co-ordinated study, wrote the manuscript and gave substantial contribution to the molecular part of the research. RS planned and co-ordinated the study, wrote the manuscript. All authors read and approved the final manuscript.

## Supplementary Material

Additional file 1 **Panther Alignment**. Multiple species alignment of the TRPV4 protein sequence on Panther shows that the amino acids we found to be mutated are all conserved among species. Click here for file

## References

[B1] Kannu P Aftimos S Mayne V Donnan L Savarirayan R Metatropic Dysplasia: Clinical and Radiographic Findings in 11 Patients Demonstrating Long-Term Natural History Am J Med Genet A 2007 143A 2512 2522 10.1002/ajmg.a.31941 17879966

[B2] Beck M Roubicek M Rogers JG Naumoff P Spranger J Heterogeneity of metatropic dysplasia Eur J Pediatr 1983 140 231 237 10.1007/BF00443368 6628444

[B3] Camacho N Krakow D Johnykutty S Katzman PJ Pepkowitz S Vriens J Nilius B Boyce BF Cohn DH Dominant TRPV4 Mutations in Nonlethal and Lethal Metatropic Dysplasia Am J Med Genet A 2010 152A 1169 1177 10.1002/ajmg.a.33392 20425821 PMC4169191

[B4] Le Quesne GW Kozlowski K Spondylometaphyseal Dysplasia Br J Radiol 1973 46 685 691 10.1259/0007-1285-46-549-685 4199241

[B5] Nural MS Diren HB Sakarya O Yalin T Dağdemir A Kozlowski type spondylometaphyseal dysplasia: a case report with literature review Diagn Interv Radiol 2006 12 70 73 16752352

[B6] Krakow D Vriens J Camacho N Luong P Deixler H Funari TL Bacino CA Irons MB Holm IA Sadler L Okenfuss EB Janssens A Voets T Rimoin DL Lachman RS Nilius B Cohn DH Mutations in the Gene Encoding the Calcium-Permeable Ion Channel TRPV4 Produce Spondylometaphyseal Dysplasia, Kozlowski Type and Metatropic Dysplasia Am J Hum Genet 2009 84 307 315 10.1016/j.ajhg.2009.01.021 19232556 PMC2667978

[B7] Shohat M Lachman R Gruber HE Rimoin DL Brachyolmia: Radiographic and Genetic Evidence of Heterogeneity Am J Med Genet 1989 33 209 219 10.1002/ajmg.1320330214 2669482

[B8] Superti-Furga A Unger S Nosology Group of International Skeletal Dysplasia Society Nosology and Classification of Genetic Skeletal Disorders: 2006 Revision Am J Med Genet A 2010 143A 1 18 10.1002/ajmg.a.31483 17120245

[B9] Rock MJ Prenen J Funari VA Funari TL Merriman B Nelson SF Lachman RS Wilcox WR Reyno S Quadrelli R Vaglio A Owsianik G Janssens A Voets T Ikegawa S Nagai T Rimoin DL Nilius B Cohn DH Gain-of-function mutations in TRPV4 cause autosomal dominant brachyolmia Nat Genet 2008 40 999 1003 10.1038/ng.166 18587396 PMC3525077

[B10] Vriens J Watanabe H Janssens A Droogmans G Voets T Nilius B Cell swelling, heat and chemical agonists use distinct pathways for the activation of the cation channel TRPV4 Proc Natl Acad Sci USA 2004 101 396 401 14691263 10.1073/pnas.0303329101 PMC314196

[B11] Mizoguchi F Mizuno A Hayata T Nakashima K Heller S Ushida T Sokabe M Miyasaka N Suzuki M Ezura Y Noda M Transient receptor potential vanilloid 4 deficiency suppresses unloading-induced bone loss J Cell Physiol 2008 216 47 53 10.1002/jcp.21374 18264976

[B12] Cameron TL Belluoccio D Farlie PG Brachvogel B Bateman JF Global comparative transcriptome analysis of cartilage formation *in vivo* BMC Dev Biol 2009 9 20 36 10.1186/1471-213X-9-20 19272164 PMC2662817

[B13] Muramatsu S Wakabayashi M Ohno T Amano K Ooishi R Sugahara T Shiojiri S Tashiro K Suzuki Y Nishimura R Kuhara S Sugano S Yoneda T Matsuda A Functional gene screening system identified TRPV4 as a regulator of chondrogenic differentiation J Biol Chem 2007 282 32158 32167 10.1074/jbc.M706158200 17804410

[B14] Dai J Kim OH Cho TJ Schmidt-Rimpler M Tonoki H Takikawa K Haga N Miyoshi K Kitoh H Yoo WJ Choi IH Song HR Jin DK Kim HT Kamasaki H Bianchi P Grigelioniene G Nampoothiri S Minagawa M Miyagawa SI Fukao T Marcelis C Jansweijer MCE Hennekam RCM Bedeschi F Mustonen A Jiang Q Ohashi H Furuichi T Unger S Zabel B Laush E Superti-Furga A Nishimura G Ikegawa S Novel and recurrent TRPV4 mutations and their association with distinct phenotypes within the TRPV4 dysplasia family J Med Genet 2010 47 704 709 10.1136/jmg.2009.075358 20577006

[B15] Kumar B Kannu P Savarirayan R Chan Y Lethal metatropic dysplasia: a case report Pathology 2007 39 177 181 10.1080/00313020601123854 17365839

[B16] Kozlowski K Beemer FA Bens G Dijkstra PF Iannaccone G Emons D Lopez-Ruiz P Masel J Van Nieuwenhuizen O Rodriguez-Barrionuevo C Spondylo-metaphyseal Dysplasia (report of 7 cases and essay of classification) Prog Clin Biol Res 1982 104 89 101 7163293

[B17] Maroteaux P Spranger J The spondylometaphyseal dysplasias. A tentative classification Pediatr Radiol 1991 21 293 297 10.1007/BF02018629 1870931

[B18] Gardner J Beighton P Brachyolmia: an Autosomal Dominant Form Am J Med Genet 1994 49 308 312 10.1002/ajmg.1320490313 8209891

[B19] Vriens J Owsianik G Janssens A Voets T Nilius B Determinants of 4 alpha-phrobol sensitivity in transmembrane domains 3 and 4 of the cation channel TRPV4 J Biol Chem 2007 282 12796 12803 10.1074/jbc.M610485200 17341586

[B20] Auer-Grumbach M Olschewski A Papić L Kremer H McEntagart ME Uhrig S Fischer C Fröhlich E Bálint Z Tang B Strohmaier H Lochmüller H Schlotter-Weigel B Senderek J Krebs A Dick KJ Petty R Longman C Anderson NE Padberg GW Schelhaas HJ Van Ravenswaaij-Arts CMA Pieber TR Crosby AH Guelly C Alterations in the ankyrin domain of TRPV4 cause congenital distal SMA, scapuloperoneal SMA and HMSN2C Nat Genet 2010 42 160 164 10.1038/ng.508 20037588 PMC3272392

[B21] Deng HX Klein CJ Yan J Shi Y Wu Y Fecto F Yau HJ Yang Y Zhai H Siddique N Hedley-Whyte ET DeLong R Martina M Dyck PJ Siddique T Scapuloperoneal spinal muscular atrophy and CMT2C are allelic disorders caused by alterations in TRPV4 Nat Genet 2010 42 165 169 10.1038/ng.509 20037587 PMC3786192

[B22] Landouré G Zdebik AA Martinez TL Burnett BG Stanescu HC Inada H Shi Y Taye AA Kong L Munns CH Choo SS Phelps CB Paudel R Houlden H Ludlow CL Caterina MJ Gaudet R Kleta R Fischbeck KH Sumner CJ Mutations in TRPV4 cause Charcot-Marie-Tooth disease type 2C Nat Genet 2010 42 170 174 10.1038/ng.512 20037586 PMC2812627

[B23] Nishimura G Dai J Lausch E Unger S Megardbané A Kitoh H Kim OH Cho TJ Bedeschi F Benedicenti F Mendoza-Londono R Silengo M Schmidt-Rempler M Spranger J Zabel B Ikegawa S Superti-Furga A Spondylo-epiphyseal dysplasia, Maroteaux type (pseudo-Morquio syndrome type 2), and parastremmatic dysplasia are caused by TRPV4 mutations Am J Med Genet A 2010 152A 1443 1449 20503319 10.1002/ajmg.a.33414

