# Supplementary figures and images for: TRPV4 related skeletal dysplasias: a phenotypic spectrum highlighted byclinical, radiographic, and molecular studies in 21 new families
Source: Orphanet J Rare Dis. 2011 Jun 9;6:37. doi: 10.1186/1750-1172-6-37 (PMC3135501; doi:10.1186/1750-1172-6-37)

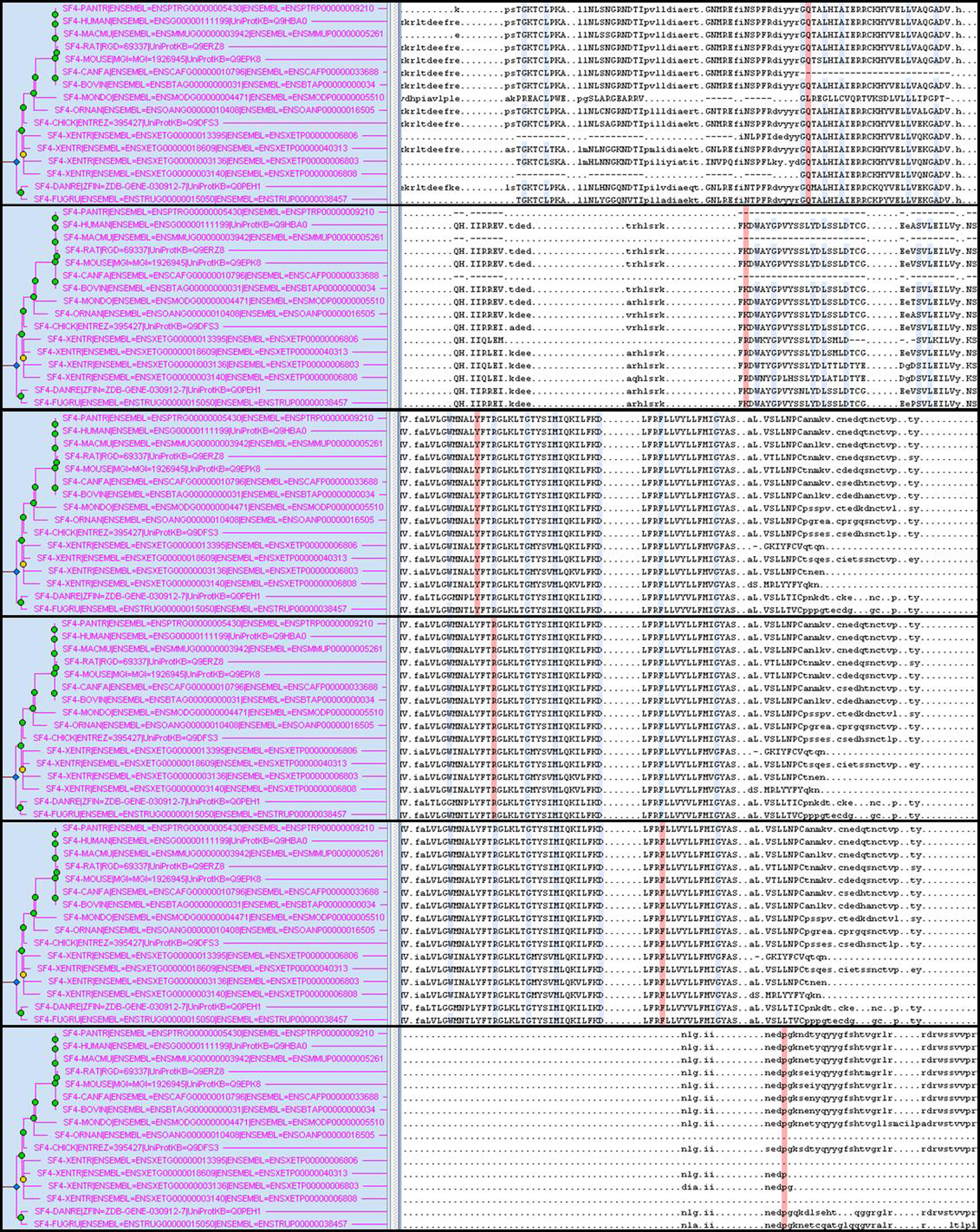

Supplement: Additional file 1 — Panther Alignment. Multiple species alignment of the TRPV4 protein sequence on Panther shows that the amino acids we found to be mutated are all conserved among species. [file 1750-1172-6-37-S1.JPEG]
